# Virtual Current Sensor in the Fault-Tolerant Field-Oriented Control Structure of an Induction Motor Drive

**DOI:** 10.3390/s19224979

**Published:** 2019-11-15

**Authors:** Michal Adamczyk, Teresa Orlowska-Kowalska

**Affiliations:** Department of Electrical Machines, Drives and Measurements, Wroclaw University of Science and Technology, Wybrzeze Wyspianskiego 27, 50-370 Wroclaw, Poland; michal.adamczyk@pwr.edu.pl

**Keywords:** electric vehicle, induction motor drive, current sensor fault, fault-tolerant control, field-oriented control, current estimator

## Abstract

Designing electrical drives resistant to the failures of chosen sensors has recently become increasingly popular due to the possibility of their use in fault-tolerant control (FTC) systems including drives for electric vehicles. In this article, a virtual current sensor (VCS) based on an algorithmic method for the reconstruction of the induction motor (IM) phase currents after current sensor faults was proposed. This stator current estimator is based only on the measurements of the DC-bus voltage in the intermediate circuit of the voltage-source inverter (VSI) and a rotor speed. This proposal is dedicated to fault-tolerant vector controlled IM drives, where it is necessary to switch to scalar control as a result of damage to the current sensors. The proposed VCS allows further uninterrupted operation of the direct rotor-field oriented control (DRFOC) of the induction motor drive. The stator current estimator has been presented in the form of equations, enabling its practical implementation in a microprocessor system. Simulation studies of the proposed algorithm in an open and closed-loop DRFOC structure are presented under different operation conditions of the drive system. The experimental verification of the proposed method is also presented and the accuracy of the stator current estimation algorithm is analyzed under various operating conditions of the drive system.

## 1. Introduction

The growing interest in electric vehicles in passenger transport (cars, trams, buses) has resulted in research into new solutions to increase the efficiency and reliability of electric drives in these applications, while reducing the size and weight. Electric motors, particularly induction (IM) and permanent magnet synchronous (PMSM) motors, are less complex than internal combustion engines and much easier to model and control [1]. In addition, well-developed theoretical and technical methods of controlling these motors, in particular the so-called vector methods, allow for very precise control of the driving torque [2,3].

Vector control of an IM requires information to be provided to the control system about state variables such as hard-to-measure stator and rotor fluxes or electromagnetic torque as well as the measurement of the voltage in the intermediate circuit of the voltage-source inverter (VSI), angular velocity, and phase currents. The most popular group of techniques for the estimation of difficult-to-measure state variables are algorithmic methods [2,3], whose accuracy is largely dependent on the correct identification of parameters of the IM equivalent circuit and the accuracy of the phase current measurement. Therefore, it is required that information on the actual values of phase currents are provided not only to the control system itself (current feedbacks in field-oriented control [3]), but also to the flux estimation module during drive operation.

Electric drives may fail like all other technical devices [4,5,6], and this damage may affect both the motors [7] and the power electronics converters (supplying motors) [8] and measuring sensors [9]. These sensors are used to obtain information about signals such as the speed, voltage, and current, used in the feedback of the drive control structure. Therefore, fault-tolerant control (FTC) strategies of electrical drives have been developed in recent years [4,6] including fault diagnosis and isolation (FDI) methods [8,10]. The main purpose of FTC is to ensure that the system works both under normal operating conditions as well as after a fault has occurred in some of its components.

In systems where cost plays a subordinate role, safety and reliability are the most important factors. Therefore, speed sensors and voltage and current sensors are also used in the electric drives of vehicles to ensure safe driving. However, due to the limited reliability of these devices, solutions should be provided that will allow for the continued operation or safe braking of the driving motor in the event of a failure of the measuring sensors. A solution may be the introduction of software redundancy in the form of virtual sensors based on state variable estimators developed on the basis of the mathematical model of the motor [2,5,6].

As above-mentioned, vector control methods of the IM drive cannot operate without actual information about the stator currents. Therefore, in the event of a failure of the current sensors, it is necessary to replace them with virtual current sensor (VCS) (i.e., systems that reconstruct stator currents based on other available measurement signals).

In the known literature, many solutions can be found regarding methods of stator phase current estimation after sensor failure, but all focus on the use of an additional current sensor in the intermediate circuit of the VSI and the use of three-phase symmetry on the phase variables. Such a concept was already proposed in the 1980s by Williams and Green [11]. In the 1990s, the inverter was additionally protected against short circuits and overloads [12,13].

Under the duration of active VSI vectors, the current in the intermediate circuit is equal to one or the sum of two phase currents, which with a symmetrical receiver, IM, allows for the determination of the current in a given phase. However, this method introduces many problems associated with the correct measurement of the DC current in the intermediate circuit. To implement it correctly, it is necessary to choose the sampling moment, so that the value of the direct current flowing through the sensor is measured in a steady state. Additionally, the dead time *T_d_*, (which protects against short circuits in VSI), the time of switching on the next sequence of VSI switches *T_on_*, the settling time *T_set_* of the signal, and the time *T_A/D_* of the analog-to-digital processing should be taken into account. Therefore, the duration of a given active vector must be long enough to allow for the estimation of a given phase current. This means that the reference voltage vector must be in the measurable area. In addition, as a general rule, the average voltage value cannot be changed in one cycle [11,12,13,14,15,16,17].

Solving the problem with immeasurable areas has been the subject of many scientific papers. One way is to introduce measurement vectors [18,19]. In [18], the measurement vector insertion method (MVIM) was proposed, which involves the introduction of a special switching sequence for VSI switches each time the reference voltage vector is in the unmeasurable area. This strategy allows for the determination of the three phase currents, but requires the introduction of additional measurement time and increases the current ripples. The introduction of additional vectors was also presented in [20], where the authors showed the possibility of reconstructing three phase currents, without additional calculation effort. The proposed algorithm, however, involves limiting the maximum speed to 86% of its rated value when compared to a classic structure. The authors of [21] presented the fast switching (FS) strategy with the idea to introduce six space vectors shifted 30 degrees relative to the active vectors. Fast switching between new vectors in the sequence proposed by the authors allows for the determination of two phase currents. Additionally, in this case, the maximum speed of the system is limited; moreover, the method is associated with increased electromagnetic moment ripples.

One way to ensure the minimum duration of active vectors is to use their phase shift [22,23], which is effective in systems with a low modulation index, where the measurable area is much smaller than the minimum time necessary for proper current sampling. In [22], the phase shift of the active vectors was implemented by the modification of PWM, while maintaining the original duration of the active vectors. However, this solution requires the introduction of a limit for the PWM modulation coefficient, so that no saturation of the modulator occurs.

Reconstruction of the phase currents in a conventional manner is possible during the duration of active vectors, because only in such a situation does one or the sum of two phase currents flow through the current sensor located in the intermediate DC circuit of VSI. During the duration of zero vectors, all the stator winding currents flow through the sensor or no current flows. As the IM is a symmetrical receiver, the sum of any three phase quantities is zero; as a consequence, in both cases, it is not possible to reconstruct the line current during the zero vectors. The studies in [24,25,26] presented the idea of placing a DC current sensor between a group of transistors in such a way that it is possible to measure the current in VSI zero states. This method is particularly effective in systems operating at low speed, where there is a low modulation rate. However, this concept requires modification of the transistor connecting cables to allow for the specific placement of a DC sensor.

The estimated phase currents obtained on the basis of a single current sensor in the DC-bus intermediate circuit of VSI contained deformations and higher harmonics. A solution to this problem when applied to low-cost systems was presented in [27], where a method of current estimation based on the mathematical model of the PMSM implemented on a signal processor (DSP) was proposed. The authors showed how to predict the current in seven steps, thanks to which the error in the current estimation under transient states was reduced. Then, they proposed a step reduction. In [28], the authors presented the half pulse shift (HPS) method, where the modified PWM signal consisted of two types of regions: one allowing for DC current sampling and the second that maintained its average value within the accepted limits. Experimental studies have confirmed its resistance to current ripple.

The phase current estimation solutions presented above require the introduction of an additional DC current sensor into the VSI intermediate circuit. In addition, most of these methods are directed to sensorless and low–cost systems equipped with only a single DC sensor. It has been shown that these methods are subject to the limitations resulting from measurable areas, the introduction of an additional sensor in DC circuit, and increase in ripples and harmonics. The solution directed to systems where there is no additional DC sensor is not shown. Therefore, in a conventional drive system with vector control structure, damage to the phase current sensors is associated with the need to switch the vector control system to the simplest scalar control type *U/f* = const (without current feedback).

This paper presents a solution that in systems with angular velocity measurement will allow for further implementation of vector control in the event of failure of all phase current sensors. The algorithm of VSC uses a popular rotor flux estimator model, and thus speed measurement is required to implement it. This method is dedicated to fault-tolerant systems, where as a result of damage to current sensors, it is necessary to switch to scalar control [9]. The proposed VCS allows further, uninterrupted operation of the direct rotor-field oriented control (DRFOC) of the induction motor drive. The stator current estimator has been presented in the form of equations to enable its practical implementation on a microprocessor system. The concept of this method was preliminarily tested in a simulation in [29], and in this paper, an experimental verification of the proposed method is shown in various operation conditions of the drive system by using digital signal processor implementation.

The rest of this paper is organized as follows. Section 2 presents the IM mathematical model and the algorithm based on it for estimating three phase currents written in a discrete form that enables practical implementation in a microprocessor. Section 3 is devoted to the simulation tests of the described method, applied to the direct rotor flux oriented control (DRFOC), while Section 4 presents the experimental verification of the proposed stator current estimation method. The research results are summarized in Section 5.

## 2. Estimation Algorithm of the Induction Motor Currents

### 2.1. Mathematical Model of the Induction Motor

The mathematical model of the IM, with commonly used simplifying assumptions [2,3]:

- the stator windings and the rotor cage are replaced by a concentric winding;
- three-phase motor symmetry and uniformity of the air gap are assumed;
- the phenomena of hysteresis, magnetic saturation, eddy currents, and anisotropy are ignored; and
- the constant resistances and winding inductance are assumed,

can be represented in a vector form (using spatial vectors) in a stationary coordinate system (*α–β*), in relative quantities, [p.u.] [3] as follows:

- voltage equation of the stator winding:
  (1) $$\frac{d}{dt}\Psi_{s}=\left(u_{s}-r_{s}i_{s}\right)\frac{1}{T_{N}}$$
- voltage equation of the rotor winding:
  (2) $$\frac{d}{dt}\Psi_{r}=\left(\frac{r_{r}}{l_{r}}\left(l_{m}i_{s}-\Psi_{r}\right)+j\omega_{m}\Psi_{r}\right)\frac{1}{T_{N}}$$
- flux–current equations:
  (3a) $$\Psi_{s}=l_{s}i_{s}+l_{m}i_{r}$$
  (3b) $$\Psi_{r}=l_{r}i_{r}+l_{m}i_{s}$$
- expression for electromagnetic torque:
  (4) tem=Im{Ψs*is}
- equation of motion:
  (5) $$\frac{d}{dt}\omega_{m}=\left(t_{em}-t_{L}\right)\frac{1}{T_{M}}$$

where **Ψ***_s_*, **Ψ***_r_* are the spatial vectors of the stator and rotor fluxes, respectively; **i***_s_*, **i***_r_* are the spatial vectors of the stator and rotor currents, respectively; **u***_s_* is the spatial vector of the stator voltage; *t_em_, t_L_* are the electromagnetic and load torques, respectively; *ω_m_* is the angular speed; *T_M_* is the mechanical time constant; *r_s_*, *r_r_* are the stator and rotor winding resistances, respectively; *l_s_* = *l_σs_* + *l_m_*, *l_r_* = *l_σr_* + *l_m_* are the stator and rotor winding inductances, respectively; *l_σs_*, *l_σr_* are the stator and rotor winding leakage inductances, respectively; *l_m_* is the main inductance; *f_sN_* is the nominal frequency; and *T_N_* = 1/2 π*f_sN_*.

The presented mathematical model assumes three-phase motor symmetry, which means that for any phase quantity *k_A_*(*t*), *k_B_*(*t*), *k_C_*(*t*), the condition is met:

(6) $$k_{A}(t)+k_{B}(t)+k_{C}(t)=0.$$

Therefore, it is possible to apply the estimation of (*α–β*) components of the stator voltage vector based on the *u_dc_* voltage in the VSI intermediate circuit and the logic states (*S_A_*, *S_B_*, *S_C_*) of the inverter connectors [2,3], assuming that they are treated as ideal switches, according to:

(7) $$u_{s\alpha }=\frac{2}{3}u_{dc}\left(S_{A}-\frac{1}{2}\left(S_{B}+S_{C}\right)\right)$$

(8) $$u_{s\beta }=\frac{\sqrt{3}}{3}u_{dc}\left(S_{B}-S_{C}\right)$$

### 2.2. Stator Current Estimation Algorithm

The algorithm for estimating three phase currents only uses the measurement of the angular velocity *ω_m_* and direct voltage *u_dc_* in the VSI intermediate circuit, on the basis of which phase voltages can be determined, according to Equations (7) and (8):

(9) $$\left[\begin{array}{c} u_{sA}\\ u_{sB}\\ u_{sC} \end{array}\right]=\left[\begin{array}{c} \frac{u_{sA}^{*}+1}{2}\\ \frac{u_{sA}^{*}+1}{2}\\ \frac{u_{sA}^{*}+1}{2} \end{array}\right]\times \left[\begin{array}{ccc} \frac{2}{3} & -\frac{1}{3} & -\frac{1}{3}\\ -\frac{1}{3} & \frac{2}{3} & -\frac{1}{3}\\ -\frac{1}{3} & -\frac{1}{3} & \frac{2}{3} \end{array}\right]\times u_{dc}$$

where the first matrix is responsible for matching the reference voltages to the PWM modulator, while the second arises from the simplified mathematical model of VSI.

The idea is based on the use of the flux–current (Equation (3)). Determining the rotor current vector **i***_r_* from Equation (3b) and substituting for (3a), we obtain:

(10) $$\Psi_{s}=l_{s}i_{s}+\frac{l_{m}}{l_{r}}\Psi_{r}-\frac{l_{m}^{2}}{l_{r}}i_{s}=\frac{l_{m}}{l_{r}}\Psi_{r}+\left(1-\frac{l_{m}^{2}}{l_{s}l_{r}}\right)l_{s}i_{s}=\frac{l_{m}}{l_{r}}\Psi_{r}+\sigma l_{s}i_{s}$$

After differentiating both sides of Equation (10):

(11) $$\frac{d}{dt}\Psi_{s}=\frac{l_{m}}{l_{r}}\frac{d}{dt}\Psi_{r}+\sigma l_{s}\frac{d}{dt}i_{s},$$

where $\sigma =1-l_{m}^{2}/l_{s}l_{r}$ is the leakage factor.

The dynamics of the stator flux vector **Ψ***_s_* (left-hand side of (11)) can be described as the difference between the stator voltage and the product of the stator resistance and current as in Equation (1), therefore, Equation (11) can be represented as follows:

(12) $$\frac{l_{m}}{l_{r}}\frac{d}{dt}\Psi_{r}+\sigma l_{s}\frac{d}{dt}i_{s}=\left(u_{s}-r_{s}i_{s}\right)\frac{1}{T_{N}}$$

Next, after transformation, the relationship describing the dynamics of the estimated stator current vector is obtained:

(13) $$\frac{d}{dt}i_{s}^{e}=\frac{1}{\sigma l_{s}}\left(u_{s}-r_{s}i_{s}^{e}-T_{N}\frac{l_{m}}{l_{r}}\frac{d}{dt}\Psi_{r}\right)\frac{1}{T_{N}}$$

For practical microprocessor implementation of the described algorithm, it should be written in a discrete form in a stationary coordinate system (*α−β*). Therefore, Equation (13) in a discrete form, using the explicit Euler method, takes the following form:

(14a) $$i_{s\alpha }^{e}(k+1)=i_{s\alpha }^{e}(k)+\frac{1}{\sigma l_{s}}\left(u_{s\alpha }(k)-r_{s}i_{s\alpha }^{e}(k)-\frac{T_{N}}{T_{s}}\frac{l_{m}}{l_{r}}\left(\Psi_{r\alpha }^{i}(k+1)-\Psi_{r\alpha }^{i}(k)\right)\right)\frac{T_{s}}{T_{N}}$$

(14b) $$i_{s\beta }^{e}(k+1)=i_{s\beta }^{e}(k)+\frac{1}{\sigma l_{s}}\left(u_{s\beta }(k)-r_{s}i_{s\beta }^{e}(k)-\frac{T_{N}}{T_{s}}\frac{l_{m}}{l_{r}}\left(\Psi_{r\beta }^{i}(k+1)-\Psi_{r\beta }^{i}(k)\right)\right)\frac{T_{s}}{T_{N}}$$

To obtain the estimated value of the stator current vector components, the rotor flux should be determined, whose components resulting from the well-known current model of the rotor flux vector (Equation (2)) were described in (*α–β*) coordinates using the symmetric Euler (SE) discretization method [30]:

(15a) $$\Psi_{r\alpha }^{i}(k+1)=\Psi_{r\alpha }^{i}(k)+\left[\frac{r_{r}}{l_{r}}\left(l_{m}i_{s\alpha }^{e}(k)-\Psi_{r\alpha }^{i}(k)\right)-\omega_{m}(k)\Psi_{r\beta }^{i}(k)\right]\frac{T_{s}}{T_{N}}\;,$$

(15b) $$\;\Psi_{r\beta }^{i}(k+1)=\Psi_{r\beta }^{i}(k)+\left[\frac{r_{r}}{l_{r}}\left(l_{m}i_{s\beta }^{e}(k)-\Psi_{r\beta }^{i}(k)\right)+\omega_{m}(k)\Psi_{r\alpha }^{i}(k+1)\right]\frac{T_{s}}{T_{N}}$$

where *T_s_* is the sampling time.

By transforming Equation (14) after substituting Equation (15), the final equations of the stator current estimator in the (*α–β*) system are obtained:

(16a) $$\begin{array}{l} i_{s\alpha }^{e}(k+1)=i_{s\alpha }^{e}(k)+\\ +\frac{1}{\sigma l_{s}}\left(u_{s\alpha }(k)-r_{s}i_{s\alpha }^{e}(k)-\frac{l_{m}}{l_{r}}\left(\frac{r_{r}}{l_{r}}\left(l_{m}i_{s\alpha }^{e}(k)-\Psi_{r\alpha }^{i}(k)\right)-\omega_{m}(k)\Psi_{r\beta }^{i}(k)\right)\right)\frac{T_{s}}{T_{N}} \end{array}$$

(16b) $$\begin{array}{l} i_{\;s\beta }^{e}(k+1)=i_{s\beta }^{e}(k)+\\ +\frac{1}{\sigma l_{s}}\left[u_{s\beta }(k)-r_{s}i_{s\beta }^{e}(k)-\frac{l_{m}}{l_{r}}\left(\frac{r_{r}}{l_{r}}\left(l_{m}i_{s\beta }^{e}(k)-\Psi_{r\beta }^{i}(k)\right)+\omega_{m}(k)\Psi_{r\alpha }^{i}(k+1)\right)\right]\frac{T_{s}}{T_{N}} \end{array}$$

**Figure 1:**
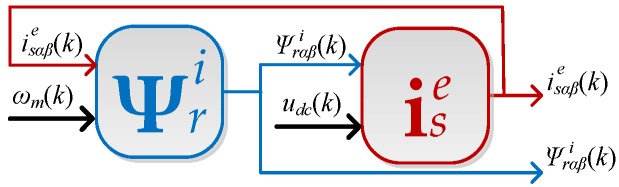
Block diagram of the stator current estimation based on the measurement of angular velocity and DC voltage in the VSI intermediate circuit.

These equations do not contain algebraic loops because $\Psi_{r\alpha }^{i}(k+1)$ is dependent on *k*-th sample, according to the SE numerical algorithm (Equation (15a)). A block diagram of the described algorithm is presented in Figure 1.

## 3. Simulation Study

### 3.1. Description of the Tested Structure and Test Scenarios

The simulation tests were carried out in two stages. In the first stage, the system was analyzed for various angular velocities in the range of ±*ω_mN_*, while in the second, the system behavior at slow start-up and reversing operation was examined. The elements of the Simscape Power System Library [31] were used to implement the VSI and IM model, and the ode1 method with step 5 × 10^−6^ was adopted for numerical calculations. The rated data and parameters of the tested IM are presented in the Appendix A (Table A1).

**Figure 2:**
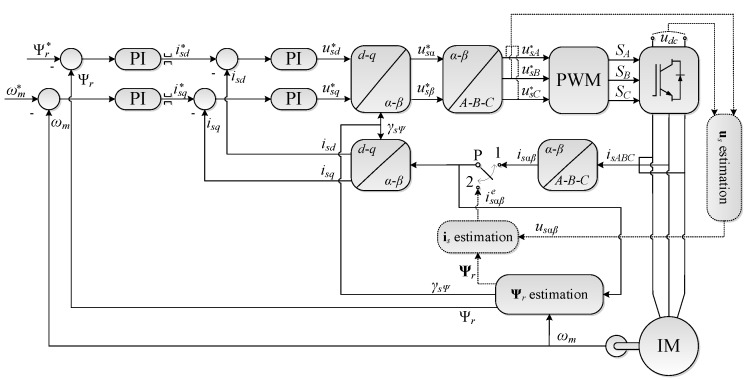
DRFOC structure with the ability to work without measuring phase currents: switch P in position 1—classical structure; switch P in position 2—structure with phase current estimation.

The system worked in the DRFOC structure using the stator currents feedbacks in the rotor flux–oriented coordinate system (*d–q*), shown in Figure 2. The simulation tests were carried out for the currents estimated using the algorithm described in Section 2 (switch P in position 2 in Figure 2).

Simulation tests were carried out in the following stages:

- the correct operation of the stator phase current estimation algorithm in a steady state of the drive operation was checked (the estimator was not included in the current feedback); and
- this algorithm was tested in various drive operating conditions, with current estimation in a closed–loop structure.

**Figure 3:**
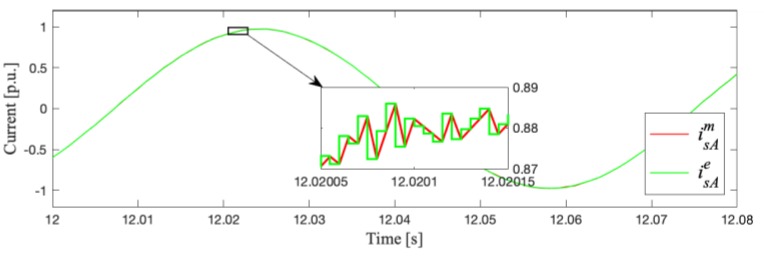
Transient of the measured and estimated current in phase A (simulation tests).

The analytical correctness of the proposed method that confirms the quality of the stator current estimation in phase A is illustrated in Figure 3. It can be seen that the proposed current estimation algorithm works very well in an open–loop (estimator not included in the DRFOC structure; switch P in position 1).

### 3.2. Analysis of the Drive System for Different Speed Trajectories in DRFOC Structure

**Figure 4:**
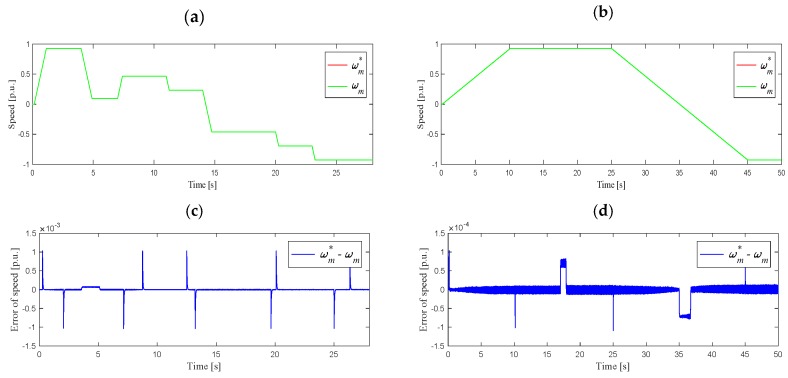
Reference and measured angular speed waveforms: (**a**) for various speed set points, (**b**) during slow start-up and reverse in the range ±*ω_mN_*, (**c**) speed error for (**a**), (**d**) speed error for (**b**).

**Figure 5:**
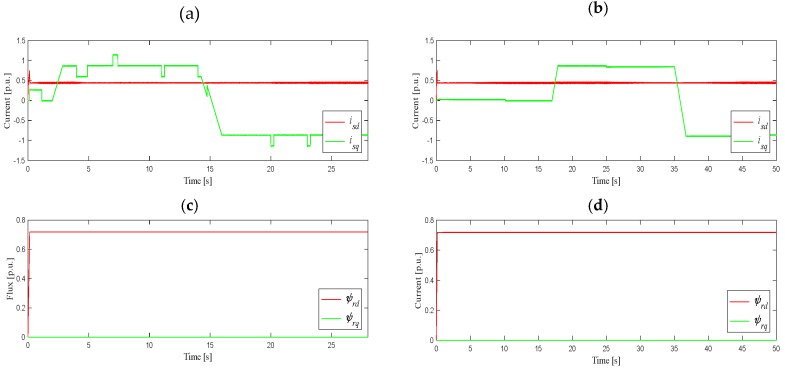
Waveforms of *d–q* components of the stator current and magnitude of the rotor flux vectors for different speed trajectories as in Figure 4a-(**a**,**c**), and in Figure 4b-(**b**,**d**).

The next stage of simulation tests was the analysis of the work on the stator current estimation algorithm in a closed DRFOC structure (switch P in position 2) for different speed trajectories, as shown in Figure 4. In the case shown in Figure 4a, the system worked at a load torque equal to 85% of its nominal value, set in 2 s, after determining the rated speed. On the other hand, slow start-up and reversing in the speed range ±*ω_mN_*, as shown in Figure 4b, were carried out as follows: start-up to the rated speed was carried out at idle-running, while reversing was carried out during the operation at 85% of the rated torque, set in 17 s. Figure 5 shows the waveforms of the *i_sd_* and *i_sq_* components of the stator current vector, and components *Ψ_rd_*, *Ψ_rq_* of the rotor flux vector, corresponding to the suitable angular speed trajectories.

As can be seen in these figures, in the presented operating conditions, the drive system worked properly, and the current estimation algorithm did not interfere with the correct operation of the vector control structure. More detailed simulation tests of the proposed stator current estimation method can be found in [23]. Next, the experimental verification based on digital signal processor implementation in the DRFOC drive system will be shown.

## 4. Experimental Results

### 4.1. Description of the Experimental Set-Up and Test Scenarios

**Figure 6:**
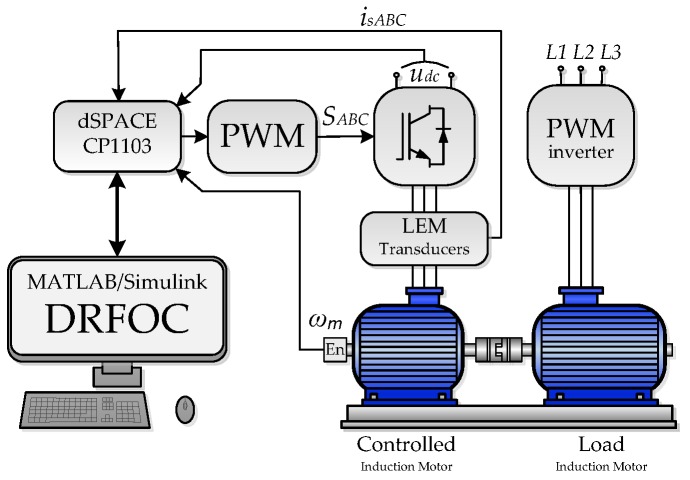
The structure of the drive system used for experimental research.

Experimental tests were realized in the drive system structure presented in Figure 6. The laboratory set–up consisted of two induction motors (1.1 kW driving motor and 1.5 kW loading motor), two VSIs, measurement devices, and the rapid prototyping system based on dSpace 1103. The whole control structure including the estimation algorithms modeled in the MATLAB/Simulink software was implemented in the DSP card. The VSI supplying the driving motor was controlled with fiber optics from dSpace, while the loading motor used its own program to control the torque. The measurement system consisted of the DC voltage sensor, three phase stator current LEM-type sensors, and the incremental encoder (5000 imp/rev.) to measure the speed.

The experimental research was divided into three stages. The first stage concerns the analysis of the stator current estimation quality in various operating conditions of the drive system; the second is devoted to the analysis of the test scenarios analogous to Section 3.2; while the last shows the system behavior when changing the P (Figure 2) switch from position 1 to position 2 and vice versa. The waveforms of the *i_sd_* and *i_sq_* components of the stator current vector obtained during the operation of the system with measured (switch P in position 1) and estimated (switch P in position 2) stator phase currents are summarized.

### 4.2. Analysis of the Stator Current Estimation in Various Operating Condition

The stator current estimation quality tests were carried out for the following cases:

1. Rated speed, no load.
2. Rated speed, 25% of the nominal value of the load.
3. Rated speed, 50% of the nominal value of the load.
4. Rated speed, 75% of the nominal value of the load.
5. Rated speed and load torque.
6. 25% of the nominal speed value, rated load.
7. 50% of the nominal speed value, rated load.
8. 75% of the rated speed, rated load.

For each of the above cases, the quality of the estimation was estimated on the basis of the average modules of the difference between the estimated and measured current in each motor phase. Since the current value was different for the adopted operating conditions, this module was divided by the average amplitude value of the phase currents to normalize the estimation error. This average error value was calculated over the ten measured periods of the stator current:

(17) $$e_{i}=\frac{1}{N}\sum_{k=1}^{N}\frac{\left|i_{sA}^{m}(k)-i_{sA}^{e}(k)\right|+\left|i_{sB}^{m}(k)-i_{sB}^{e}(k)\right|+\left|i_{sC}^{m}(k)-i_{sC}^{e}(k)\right|}{\max(i_{sA}^{m})+\max(i_{sB}^{m})+\max(i_{sC}^{m})},$$

where *N* is the number of current samples.

The test results are presented in Table 1. As can be seen from this table, the current value in the real system was estimated with high accuracy. The algorithm worked better while the system was loaded.

**Figure 7:**
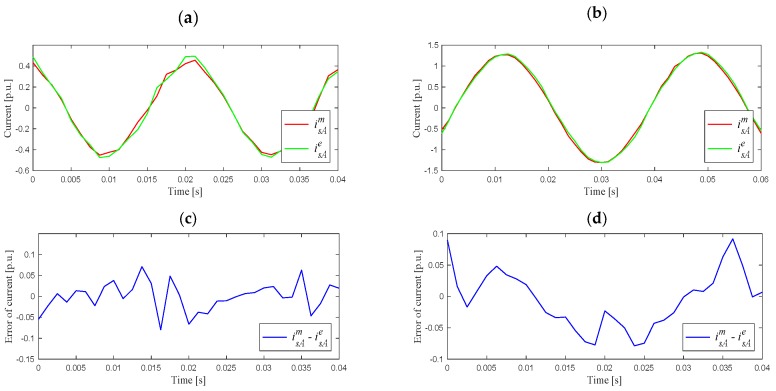
Measured and estimated current in phase A for the first case (**a**) and the seventh case (**b**), differences between the measured and estimated currents: (**c**) for (**a**), (**d**) for (**b**).

Figure 7 shows examples of the estimated and measured current waveforms for the least (1) and most (7) exact cases on the example of motor phase A.

In the case of idle–running and under a nominal load torque, the maximal differences between the measured and estimated stator current values were in the range ±0.09 [p.u.], which, however, gave slightly bigger relative errors under small load torques.

### 4.3. Analysis of the Current Estimation for Different Speed Trajectories

**Figure 8:**
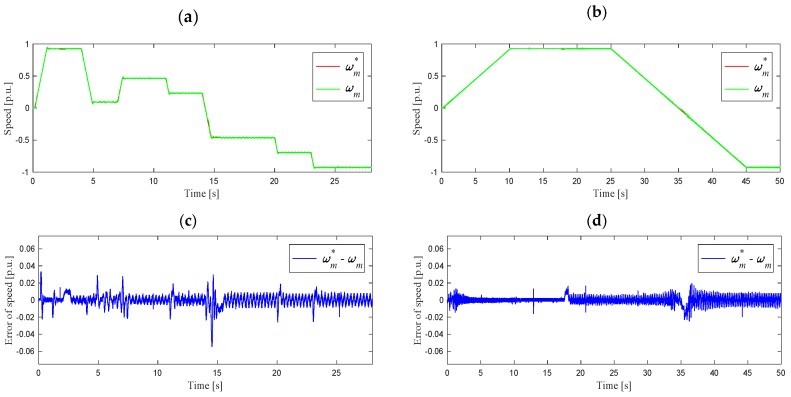
Reference and measured angular speed waveforms: (**a**) for various speed set points, (**b**) during slow start-up and reverse in the range ±*ω_mN_, t_L_* = 0.85 *t_LN_*, (**c**) speed error for (**a**), (**d**) speed error for (**b**).

**Figure 9:**
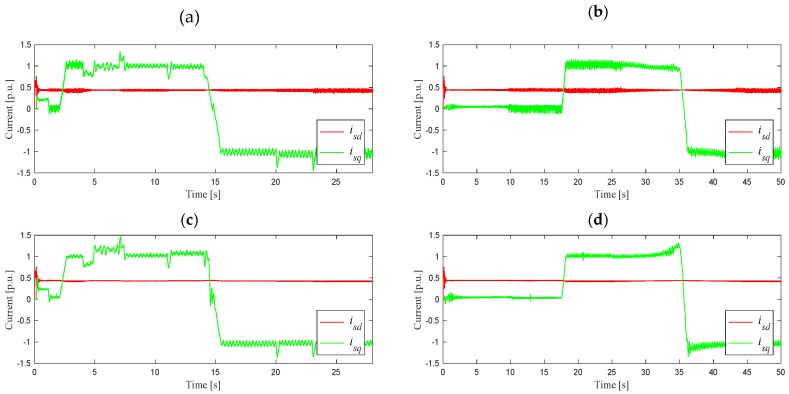
Waveforms of *d–q* components of the stator current for speed trajectories as seen in Figure 8a,b; measured currents (switch P in position 1) (**a**,**b**); estimated currents (switch P in position 2) (**c**,**d**).

For conditions analogous to those in Section 3.1, experimental studies of the system operation were carried out for various values of angular speed with a motor load of 85% of the nominal value. The angular velocity waveforms during the operation of the system with feedbacks from the estimated currents are shown in Figure 8. The next figure shows a comparison of the *i_sd_* and *i_sq_* components of the stator current vector for the measured current (Figure 9a,b) and the estimated current (Figure 9c,d).

Based on the presented results of the experimental research in various drive operating states, it can be seen that the waveforms of the stator current components during operation with the use of the measured and estimated currents were similar. The angular velocity in both cases closely followed the given trajectory. The waveforms of the *i_sd_* and *i_sq_* components were characterized by slightly smaller disturbances when switch P was in position 2 (estimated currents). An increase in the *i_sq_* value when the speed is passing slowly through the zero point could also be noted. This phenomenon does not occur when working with the measured currents, however, this does not affect the quality of the system operation (see Figure 8b).

Analysis of the above waveforms showed that the system was working properly after a failure of the stator current sensors and also when operating with a nominal load in the range of speed changes ±*ω_mN_*.

The experimental studies confirmed the analytical correctness of the proposed method of stator current estimation after current sensor failures. As it results from the quality analysis of the stator current estimation, this method is accurate, especially when the system is working with a load torque. Analysis of the system behavior in various operating states showed high efficiency of the developed stator current estimator. The system was fully controlled for the work points tested. In addition, a practical microprocessor implementation of the proposed algorithm was presented.

**Figure 10:**
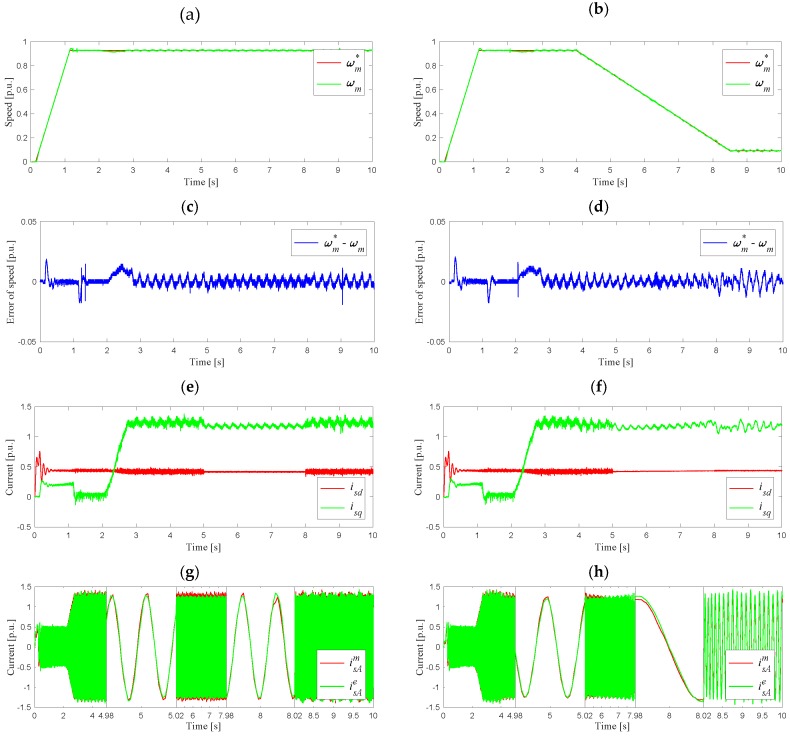
Waveforms of the motor variables under operation mode switching (time *t* = 5 s, switch P changes position from 1 to 2; time *t* = 8 s, switch P changes position from 2 to 1) for constant motor speed (**a**,**c**,**e**,**g**) and under speed decreasing (**b**,**d**,**f**,**h**): (**a**,**b**) reference and measured speeds; (**c**,**d**) speed error; (**e**,**f**) components of stator current vector; (**g**,**h**) stator current in phase A (with zooms).

The last stage of the study was the analysis of the system during a sudden change in the operating mode of the classical control structure system (switch P in position 1) to the structure with feedback from the estimated currents (switch P in position 2) and vice versa. The waveforms of speed, speed error, and currents *i_sd_*, *i_sq_, i_sA_* are shown in Figure 10.

As can be seen, during the steady state of the system as well as the dynamic state under the speed decrease, the system remained stable both when switching to the operating mode with the estimated stator currents, and after returning to the operating mode with the current measurement. Changing the operating mode did not affect the quality of the speed trajectory, which can be seen in Figure 10a,d. Due to the lack of measuring noise from the LEM current sensors, the *i_sd_* and *i_sq_* components of the stator current vector were characterized by a less oscillating waveform during operation after a failure. In the example of phase A, it was shown that the phase current waveform did not change rapidly under the operation mode switching, as shown in Figure 10g,h. It can be observed that the operation was smooth and stable, as the VCS (stator current estimator) operates all the time (“in the background”) when the real current sensor is included in the feedback loop of the DRFOC drive. Under the simulated sensor fault, the current estimator algorithm was switched-on with its initial conditions close to the real current value just before the fault occurrence. Thus, the problem of estimator convergence does not affect the drive system operation under transition of the switch P from position 1 to position 2, and vice versa.

The application of the developed method enables the implementation of the IM vector control after a stator phase current sensor failure, without losing the quality of work and without switching the drive to a scalar control in an open system (such as in [4]) or placing an additional current sensor in the DC intermediate circuit of the voltage inverter.

## 5. Conclusions

According to the presented research, the proposed method of the stator current reconstruction based on the DC bus voltage and rotor speed is effective in drive systems with induction motors equipped with a speed sensor, where safety and reliability are more important than costs. Its main advantage is the lack of restrictions, hardware modifications, and problems that occur in solutions known in the literature, because this algorithm is easy to implement, based on a popular current rotor flux model and does not need other sensors.

The developed stator current estimator can work in any IM drive system of an electric or hybrid vehicle as a VCS based on the well-known mathematical model of the electric motor. The algorithm will therefore work perfectly as additional protection of the drive system after the failure of the stator current sensors. It not only ensures the safe operation of the drive, but also allows for continued control with the vector method, even in a structure using information about the stator currents in feedback loops.

The experimental results show that VCS works very well in the DRFOC structure during a sudden change of the drive system operating mode (from normal to post-fault mode), both in the steady state of the drive system and when the rotor speed is changing. The IM can be controlled smoothly after VCS activation. This is very important in FTC solutions, because when the current sensor fails, further effective and safe work is possible regardless of the state of the drive system.

The developed VCS presents better accuracy when the IM works with the load torque. However, this is not a problem in application in electric vehicles because their propulsion system always operates with a load. For electric vehicles, this is just their mass and additional wares or people mass (when vehicles are used in transport) and landform (for example, hills).

Further research will focus on optimizing the VCS algorithm’s accuracy and its implementation in drive systems with other AC (alternative current) motors.

## Figures and Tables

**Table 1:** Quality indicators of the stator current estimation.

Test (Case)	(1)	(2)	(3)	(4)	(5)	(6)	(7)	(8)
*e_i_* [%]	7.998	6.726	4.472	3.282	5.501	4.134	3.021	3.491

## Appendix A

**Table A1:** Rated parameters of the IM tested in the simulation and experiments.

Parameter	[ph.u.]	[p.u.]
Rated phase voltage *U_N_*	230 V	0.707
Rated phase current *I_N_*	2.5 A	0.707
Rated power *P_N_*	1.1 kW	0.638
Rated speed *n_N_*	1390 rpm	0.927
Rated frequency *f_sN_*	50 Hz	-
Number of pole pairs *p_b_*	2	-
Stator winding resistance *R_s_*	5.114 Ω	0.556
Rotor winding resistance *R_r_*	5.064 Ω	0.550
Stator leakage inductance *L**_σs_*	31.6 mH	0.1079
Rotor leakage inductance *L**_σr_*	31.6 mH	0.1079
Main inductance *L_m_*	478 mH	1.6323
Mechanical time constant *T_M_*	0.25 s	-
